# Prognostic value of IGFBP2 in various cancers: a systematic review and meta‐analysis

**DOI:** 10.1002/cam4.4680

**Published:** 2022-05-11

**Authors:** Biao Zhang, Chao‐Qun Hong, Yu‐Hao Luo, Lai‐Feng Wei, Yun Luo, Yu‐Hui Peng, Yi‐Wei Xu

**Affiliations:** ^1^ Department of Clinical Laboratory Medicine Cancer Hospital of Shantou University Medical College Shantou China; ^2^ Precision Medicine Research Center, Shantou University Medical College Shantou China; ^3^ Guangdong Esophageal Cancer Institute the Cancer Hospital of Shantou University Medical College Shantou China; ^4^ Provincial Key Laboratory of Guangdong Breast Cancer Diagnosis and Treatment Cancer Hospital of Shantou University Medical College Shantou China

**Keywords:** cancer, insulin‐like growth factor binding protein 2 (IGFBP2), overall survival, prognosis

## Abstract

**Background:**

The prognostic significance of insulin‐like growth factor binding protein 2 (IGFBP2) expression has been explored in plenty of studies in human cancers. Because of the controversial results, the meta‐analysis was carried out to evaluate the relevance of IGFBP2 expression with the prognosis in various tumors.

**Methods:**

The data searched from four databases (Pubmed, Embase, Cochrane library, and Web of science) was used to calculate pooled hazard ratios (HRs) in this meta‐analysis. Subgroup analyses were stratified by ethnicity, cancer type, publication year, Newcastle–Ottawa Scale score, treatments, and populations.

**Results:**

Twenty‐one studies containing 5560 patients finally met inclusion criteria. IGFBP2 expression was associated with lower overall survival (HR = 1.57, 95% CI = 1.31–1.88) and progression‐free survival (HR = 1.18, 95% CI = 1.04–1.34) in cancer patients, but not with disease‐free survival (HR = 1.50, 95% CI = 0.91–2.46) or recurrence‐free survival (HR = 1.50, 95% CI = 0.93–2.40). The subgroup analyses indicated IGFBP2 overexpression was significantly correlated with overall survival in Asian patients (HR = 1.42, 95% CI = 1.18–1.72), Caucasian patients (HR = 2.20, 95% CI = 1.31–3.70), glioma (HR = 1.36, 95% CI = 1.03–1.79), and colorectal cancer (HR = 2.52, 95% CI = 1.43–4.44) and surgery subgroups (HR = 1.97, 95% CI = 1.50–2.58).

**Conclusion:**

The meta‐analysis showed that IGFBP2 expression was associated with worse prognosis in several tumors, and may serve as a potential prognostic biomarker in cancer patients.

## Graphical abstract

The prognostic significance of Insulin‐like growth factor binding protein 2 (IGFBP2) expression has been explored in plenty of studies in human cancers. However, the results remain controversial. Here, we conducted a systematic review and meta‐analysis to assess the relevance of IGFBP2 expression with the prognosis in various tumors.

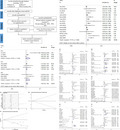

## 1 INTRODUCTION

Cancer has become a severe public problem and ranks as the main cause of the death. It was estimated that there were 18.1 million new cancer cases and 9.6 million new cancer deaths in 2018.^1^ Therefore, it has brought a heavy social burden. Although the great advances in cancer treatment technology for decades, over survival for patients of many cancer types is still low.^2^ Of note, clinical evaluation of the prognosis for many cancers mainly depends on tumor/node/metastasis (TNM) stage traditionally. Although the TNM staging is recognized as the gold standard for evaluating the survival and prognosis of cancer patients, the prognosis in cancer patients with the same TNM stage may be quite different.^3^ It would lead to the inability to choose a more suitable treatment plan for cancer patients. Tumor markers are produced by tumor cells during their growth and proliferation and play an important role in diagnosis, treatment, prognosis, and monitoring of cancer patients.^4^, ^5^ At present, many tumor markers have been used to evaluate the clinical prognosis such as CEA, CA19‐9, AFP.^6^, ^7^, ^8^ The CEA and CA19‐9 must be checked in The Chinese Colorectal Cancer Diagnosis and Treatment Standard (2017 Edition). Due to the progress of molecular biology research methods in recent years, new prognostic biomarkers have emerged one after another, which provide a more quantitative basis for prognostic evaluation and provide clinical basis for individualized treatment plan.^9^

Studies have demonstrated that the IGFBP family (IGFBP1–6) are relevant in many cancers.^10^ The presence of different IGFBPs as prognostic factors have also been reported. A literature‐based survey clearly shows that the association between IGFBP2–3 and cancer was widely studied, while less studies were done on the remaining IGFBP family members (IGFBP1, IGFBP4, IGFBP5, and IGFBP6). For IGFBP3, the most extensively studied molecule among the IGFBP family in cancer, several meta‐analyses have been carried out to evaluate its prognostic value.^11^, ^12^, ^13^, ^14^ But few such assessments of IGFBP2 were done. Thus, there is an urgent need of a focused meta‐analysis of the role of IGFBP2 in cancer prognosis. As a secreted protein, insulin‐like growth factor binding protein 2 (IGFBP2) is a key member in the IGFBP family and an extracellular regulator of insulin‐like growth factor I (IGF‐I) and II (IGF‐II). IGFBP2 mediates IGF‐independent tumorigenesis by participating in intracellular and nuclear regulatory networks. Recently, increasing studies have indicated that IGFBP2 plays a critical effect on tumorigenesis through modulation of some cancer characteristics.^15^, ^16^ Moreover, IGFBP2 overexpression is correlated with tumor cell proliferation, invasion, and migration.^17^, ^18^, ^19^, ^20^, ^21^

IGFBP2 was found to be highly expressed in many cancers, including glioma,^22^, ^23^, ^24^, ^25^, ^26^, ^27^ lung cancer,^28^, ^29^, ^30^, ^31^, ^32^ colorectal cancer,^33^, ^34^ ovarian cancer (OC),^35^, ^36^ rhabdomyosarcoma,^37^ gastric cancer,^38^ breast cancer,^39^ bladder cancer,^40^ prostate cancer,^41^ endometrial cancer,^42^ penile squamous cell carcinoma,^43^ and hepatocellular carcinoma.^44^ In addition, the overexpression was found to contribute to poor prognosis in lung cancer, gastric cancer, and glioma.^25^, ^38^, ^45^ However, Zheng et al indicated that the there was no significant association between IGFBP2 and OC.^36^ Moreover, we got the hazard ratio (HR) with corresponding 95% CI (1.03, 0.14–7.82) from Kaplan–Meier (KM) curve of Chao et al, which also suggested the insignificant association in endometrial cancer.^42^ Because these insignificant results were also observed, the dependability of IGFBP2 as a prognostic biomarker in various tumors has not been reached consensus and remains controversial. Although a previous meta‐analysis study assessed the connection between IGFBP2 expression and survival in glioma patients,^46^ its research score is relatively limited. To estimate the prognostic value of IGFBP2 in various cancers better, it is necessary to perform a comprehensive meta‐analysis using data obtained from the published studies.

Therefore, we performed the meta‐analysis to assess the association between IGFBP2 expression and the survival outcomes and the prognostic significance of the IGFBP2 in cancer patients, searched from 21 literature studies containing 5560 patients.

## 2 MATERIALS AND METHODS

The study was carried out according PRISMA Guidelines.^47^ In addition, we registered a protocol with the International Prospective Register of Systematic Reviews–PROSPERO (Registration No. CRD42021240319).

### 2.1 Search strategy

A comprehensive and accurate search of articles was performed by Pubmed, Embase, Cochrane Library, and Web of Science up to February, 2021. The search strategy is shown in Supplementary Materials. In addition, we also checked the references of other relevant articles for supplementary eligible studies.

### 2.2 Eligibility criteria

Inclusion criteria included studies that: (1) explored the connection between IGFBP2 expression level and prognosis in various cancer patients; (2) provided sufficient data by table or KM curve; and (3) were published in English. Exclusion criteria included: (1) duplicated publications; (2) animal studies, cell line experiments, reviews, letters or case reports; and (3) without available or useable data.

### 2.3 Data collection and quality evaluation

The data collection and quality evaluation were completed by two researchers independently. Any disagreement would be discussed and solved by group discussion. The following data were extracted from eligible studies by a standardized information collection: first author's name, the time of publication, author's country, the gender of patient, the age of patient, the clinical stage, the type of cancer, the method of detection, outcome, study quality, and follow‐up months. Because of different definition methods in included studies and expression levels in various cancers, IGFBP2 overexpression in this study was defined according to individual definitions from the included studies. The quality of each study was evaluated by the Newcastle–Ottawa Scale (NOS). At the same time, if the NOS score was ≥7, the study would be recognized as high quality.

### 2.4 Statistical analysis

Analyses of all data were completed using Stata 12.0 software. The strength of the connection between IGFBP2 expression and prognostic items (e.g., overall survival [OS], disease‐free survival [DFS], recurrence‐free survival [RFS], and progression‐free survival [PFS]) of the cancer patients was evaluated with the HRs and the corresponding 95% CI. In addition, the Higgin's *I* ^2^ statistics was used to assess the power of heterogeneity. An *I* ^2^ value of 0% means no observed heterogeneity, with larger *I* ^2^ values meaning enhanced heterogeneity. The pooled HR was calculated by random‐effect model when the *p*‐value was <0.05 and *I* ^2^ was >50%; otherwise, a fixed‐effect model was used. Subgroup analyses stratified by cancer type, ethnicity, NOS score, publication year, treatments, and populations were carried out to further evaluate the general sources of heterogeneity. Besides, sensitivity analysis was conducted to assess the constancy of each result in this meta‐analysis. Moreover, if publication bias was detected, the number of supposedly unpublished studies to adjust for publication bias was evaluated with a trim and fill method.^48^

## 3 RESULTS

### 3.1 Study selection and study characteristics

**FIGURE 1:**
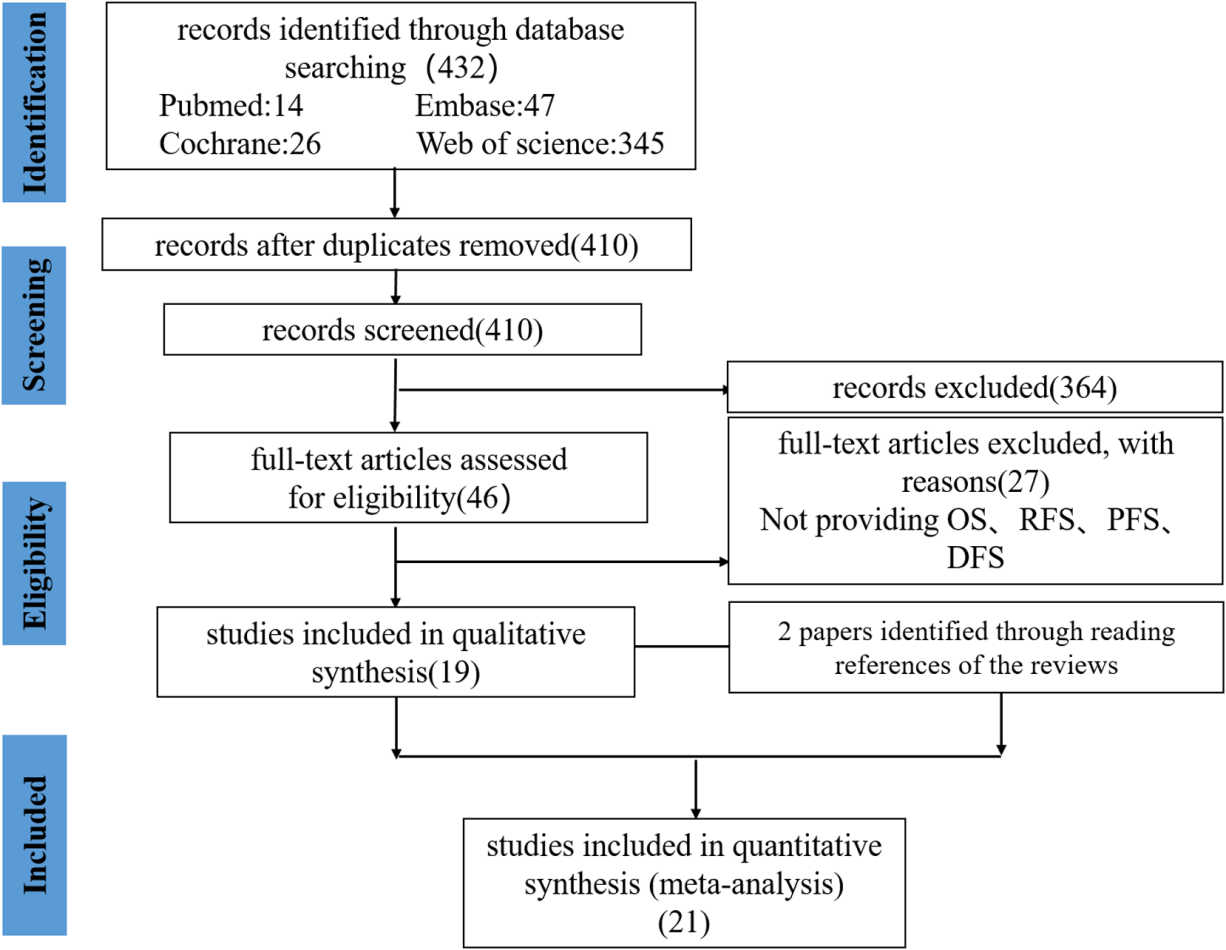
Flow diagram of study selection

We could understand the process of study selection from Figure 1. A total of 432 articles were identified using search strategy by above four datasets. Because of the duplicated articles, 22 articles were excluded. Among the remaining 410 articles, 364 articles were also removed after further reading titles and abstracts, which were chief reasons for exclusion, review, animal or cell experiments, conference abstracts, not focusing on this topic, and duplication. Twenty‐seven articles were excluded by reading full‐text articles, because of not providing OS, DFS, PFS, and RFS. Therefore, finally, there were 19 articles after evaluation of the full‐text article in total. Besides, we added two articles which were manually searched from the reference lists of published reviews.^26^, ^35^ Ultimately, 21 articles met our inclusion criteria in this study.

The primary feathers of all articles included in this study are summarized in Table 1. Twenty‐one articles comprising 5560 patients (28–1926 per study) identified in this meta‐analysis were from China, Australia, Iran, Korea, France, Italy, Japan, the Czech Republic, and Canada. The types of cancers included glioblastoma multiforme (GBM; 6 articles), lung cancer (3 articles), colorectal cancer (3 articles), ovarian cancer (OC; 2 articles), urinary system cancers (2 articles), and other cancers (5 articles). Among 21 articles, HR for OS was reported in 17 articles, and HRs of DFS, RFS, PFS were reported in 9 articles on average. The IGFBP2 expression level was detected through western blot (WB), immunohistochemistry (IHC), and quantitative real‐time polymerase chain reaction (RT‐qPCR). Other clinical parameters were about patient number (male, female, total), age, clinical stage, IGFBP2 expression level, NOS score (0–9), and follow‐up time (0.8–57.5 months). When the NOS score was greater than or equal to 7 in included articles, it was regarded as a high‐quality study. Otherwise, it was deemed low quality (Table 1). The median of NOS score was 7 (IQR: 6–7) in the included studies, ranging from 5 to 8, and the number of high and low NOS quality studies were 14 and 7, respectively.

**TABLE 1:** Main characteristics of included studies

Study	Country	Cancer type	Patient(n) F/M/T	Age	Clinical Stage (I + II/III + IV)	Detection method	Outcomes	NOS	IGFBP2expression High/low	Follow‐up (months)
Crick 2018	Denmark	Breast cancer	NA/NA/105	55	NA	NA	DFS	7	46/59	NA
Han 2014	China	GBM	50/33/83	56.7	NA	Elisa	OS	6	41/42	13.8 (0.9–34.8)
Hesam 2016	Iran	GBM	NA/NA/28	56.1	NA	Elisa/IHC	OS	7	21/7	13.25 (0.8–36.2)
Hu 2019	China	PSCC	NA/NA/56	57	NA	WB	DFS	7	18/38	26
Hur 2016	Korea	Gastric cancer	58/60/108	61	48/70	Elisa	OS	7	94/24	57.5
Li 2017	China	GBM	45/32/77	54.6	NA	RT‐qPCR	OS	7	55/22	NA
Jaime 2014	France	GBM	63/48/111	61.3	NA	Elisa	OS	6	NA	NA
Elena 2020	Italy	RMS	58/56/114	6.4	12/98	RT‐qPCR Elisa	OS	6	NA	NA
Yuan 2019	China	GBM	112/68/180	NA	NA	RT‐qPCR IHC	DSS/OS	7	43/137	NA
Guo 2013	China	Lung Cancer	71/93/164	52	46/107	Elisa	OS	7	54/29	37.5
Guo 2019	China	HCC	NA/NA/165	NA	NA	IHC	OS	6	86/79	NA
LIOU 2010	Taiwan	CRC	93/69/162	65.4	83/79	Elisa	OS	7	NA	NA
Zheng 2020	China	OC	NA/NA/1657	NA	NA	NA	OS/PFS	7	1029/627	NA
Wang 2019	China	NSCLC	NA/NA/1926	NA	NA	NA	0S	7	963/963	NA
HIDEAKI 2004	Japan	Bladder cancer	79/18/87	68.3	NA	RT‐qPCR	RFS	6	NA	NA
Brant 2005	Canada	Prostate cancer	NA/NA/82	63.2	NA	IHC	DFS	5	18/54	NA
Chao 2018	Taiwan	Endometrial cancer	NA/NA/110	NA	NA	IHC	OS	5	97/13	NA
MICHAL 2019	Czech	CRC	60/37/97	62	NA	Elisa	OS	7	12/34	NA
Tang 2018	China	Lung cancer	60/37/97	64	46/38	RT‐qPCR WB	OS	7	NA	NA
Sally 2004	Aus	OC	NA/NA/99	64	16/83	RIA	OS/PFS	8	NA	32(15–34)
Lin 2008	China	GBM	36/16/52	42.9	NA	IHC/Elisa	DFS	8	31/21	NA

Abbreviations: CRC, colorectal cancer; DFS, disease‐free survival; F, Female; GBM, glioblastoma multiforme; HCC, hepatocellular carcinoma; IGFBP2, insulin‐like growth factor binding protein 2; IHC, immunohistochemistry; M, male; NA, not available; NOS, Newcastle–Ottawa Scale; NSCLC, non‐small cell lung cancer; OC, ovarian cancer; OS, overall survival; PFS, progression‐free survival; PSCC, penile squamous cell carcinoma; RFS, recurrence‐free survival; RIA, radioimmunoassay; RMS, rhabdomyosarcoma; RT‐qPCR, quantitative real time polymerase chain reaction; T, total; WB, western blot.

### 3.2 Association between IGFBP2 expression and prognosis

**FIGURE 2:**
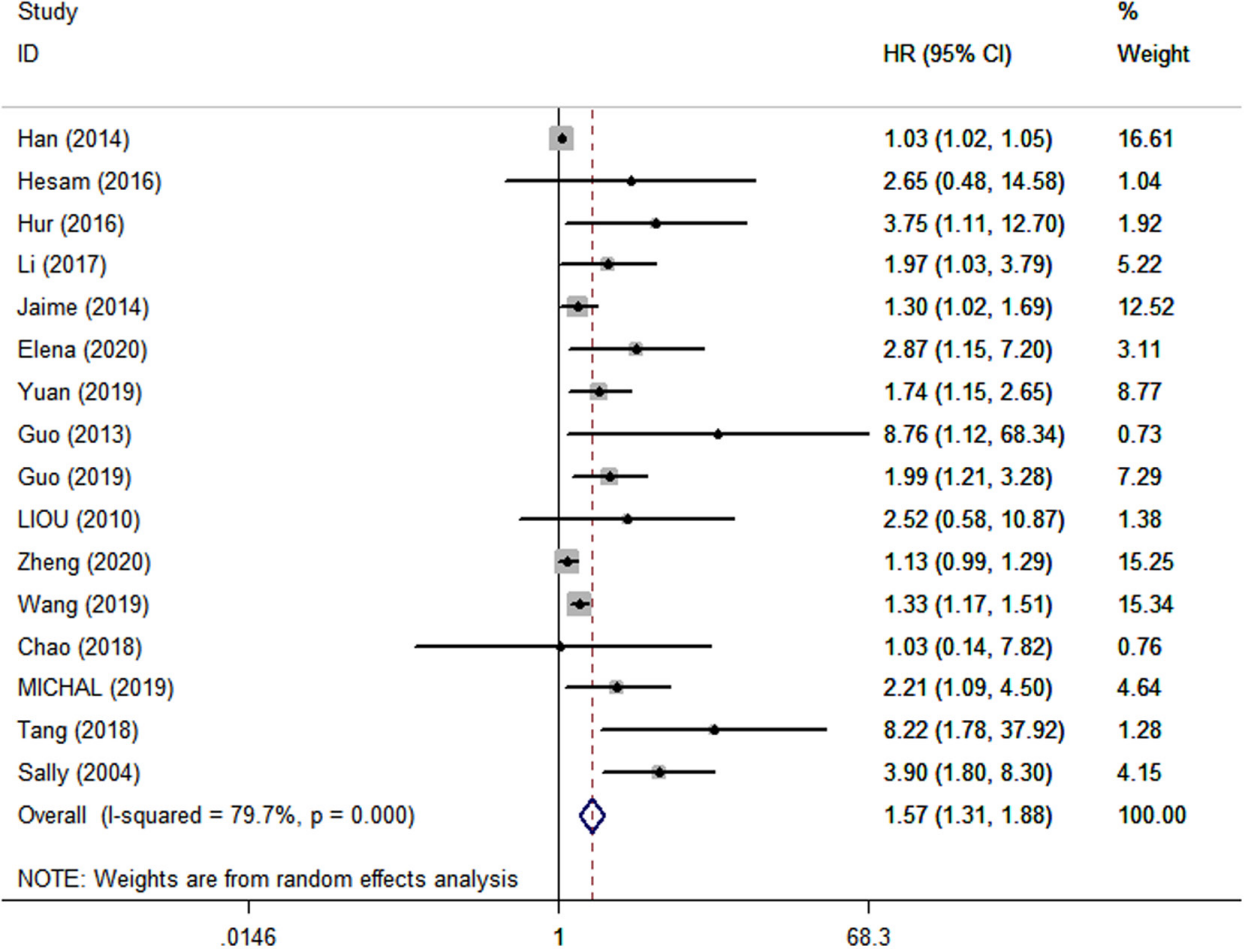
Forest plot showing hazard ratio of IGFBP2 expression for overall survival. Hazard ratios and 95% confidence interval are represented by squares and horizontal line crossing the square, respectively. The diamonds represent the pooled HR and 95% CI. All statistical tests were two‐sided.

**FIGURE 3:**
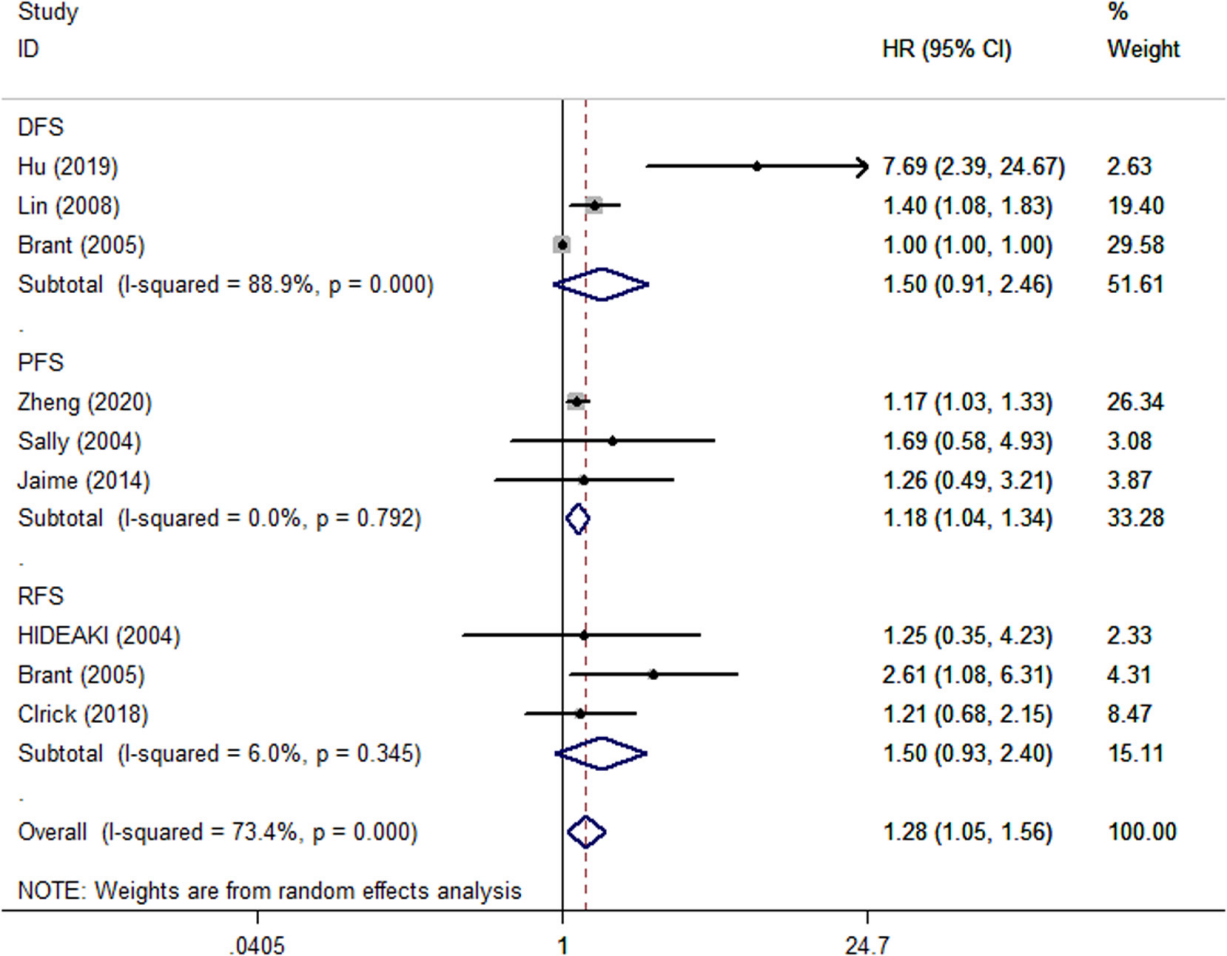
Forest plot showing hazard ratio of IGFBP2 expression for DFS, RFS, and PFS. Hazard ratios and 95% confidence interval are represented by squares and horizontal line crossing the square, respectively. The diamonds represent the pooled HR and 95% CI. All statistical tests were two‐sided.

Data for the association between IGFBP2 expression and various cancer prognosis are shown in Figures 2 and 3. A total of 17 studies were used to analyze pooled HRs for OS. High IGFBP2 expression was significantly associated with lower OS (HR = 1.57, 95% CI = 1.31–1.88) with a random‐effect model and PFS (HR = 1.18, 95% CI = 1.04–1.34) with a fixed‐effect model, compared with low IGFBP2 expression. However, high expression of IGFBP2 did not appear to be associated with DFS (HR = 1.50, 95% CI = 0.91–2.46) with a random‐effect model and RFS (HR = 1.50, 95% CI = 0.93–2.40) with a fixed‐effect model.

### 3.3 Correlation of IGFBP2 expression with clinical characteristics and subgroup analyses

**FIGURE 4:**
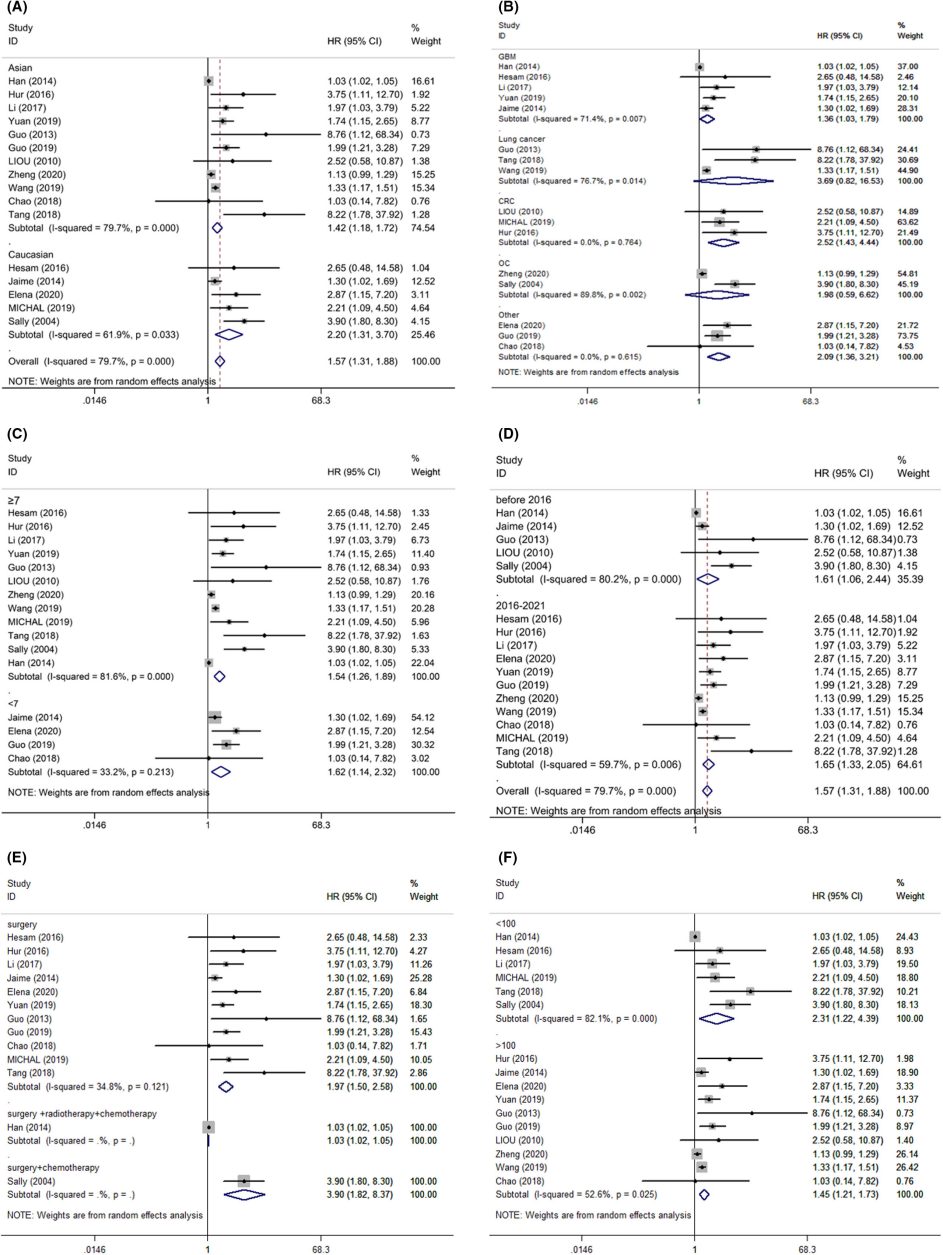
Forest plot showing hazard ratio of IGFBP2 expression for overall survival by subgroups based on (A) ethnicity, (B) cancer type, (C) NOS score, (D) publication year, (E) treatments, (F) populations. Hazard ratios and 95% confidence interval are represented by squares and horizontal line crossing the square, respectively. The diamonds represent the pooled HR and 95% CI. All statistical tests were two‐sided.

To explore the source of heterogeneity of OS, subgroup analyses of OS was performed. The major results of this subgroup analyses are shown in Table 2. The prognostic value of IGFBP2 for OS was not altered by subgroup analyses where studies were regrouped according to cancer types, ethnicity, publication year, NOS score, treatments, and populations. There were significantly worse prognosis in Asian patients (HR = 1.42, 95% CI = 1.18–1.72), Caucasians (HR = 2.20, 95% CI = 1.31–3.70) (Figure 4A), NOS score ≥7 (HR = 1.54, 95% CI = 1.26–1.89), NOS score <7 (HR = 1.62, 95% CI = 1.14–2.32) (Figure 4C), before 2016 (HR = 1.61, 95% CI = 1.06–2.44), 2016–2021 (HR = 1.65, 95% CI = 1.33–2.05) (Figure 4D), surgery (HR = 1.97, 95% CI = 1.50–2.58) (Figure 4E), populations<100 (HR = 2.31, 95% CI = 1.22–4.39), and populations >100 (HR = 1.45, 95% CI = 1.21–1.73) (Figure 4F) subgroups. Furthermore, the elevated IGFBP2 level had a poor outcome for OS according to cancer type, especially in GBM (HR = 1.36, 95% CI = 1.03–1.79), CRC (HR = 2.52, 95% CI = 1.43–4.44), and other cancers (HR = 2.09, 95% CI = 1.36–3.21) (Figure 4B). Regarding clinical characteristics, as shown in Figures S1–S3, high IGFBP2 expression was significantly correlated with age (HR = 2.09, 95% CI = 1.04–4.21) and larger tumor size (HR = 1.03, 95% CI = 1.02–1.04), but not sex (HR = 1.12, 95% CI = 0.69–1.84). In addition, the heterogeneity among ethnicity, publication year and populations subgroups were still high, but had a decrease in NOS score <7, CRC and surgery subgroups.

**TABLE 2:** Prognostic value of IGFBP2 expression for OS in cancer patients

Variables	No of studies 16	Model	Pooled HR	Heterogeneity	
				*P* value	*I* ^2^(%)
Cancer type					
GBM	5	Random	1.36 (1.03–1.79)	0.007	71.4%
Lung cancer	3	Random	3.69 (0.82–16.53)	0.014	76.7%
CRC	3	Fixed	2.52 (1.43–4.44)	0.764	0%
OC	2	Random	1.98 (0.59–6.62)	0.002	89.8%
Other	3	Fixed	2.09 (1.36–3.21)	0.615	0%
Ethnicity					
Asian	11	Random	1.42 (1.18–1.72)	0.000	79.7%
Caucasian	5	Random	2.20 (1.31–3.70)	0.033	61.9%
NOS score					
≥7	12	Random	1.54 (1.26–1.89)	0.000	81.6%
<7	4	Fixed	1.62 (1.31–1.88)	0.213	33.2%
Publication year					
Before 2016	5	Random	1.61 (1.06–2.44)	0.000	80.2%
2016–2021	11	Random	1.65 (1.33–2.05)	0.006	59.7%
Treatments	5	Random	1.61 (1.06–2.44)	0.000	80.2%
Surgery	11	Fixed	1.97 (1.50–2.58)	0.121	34.8%
Surgery+chemoradiotherapy	1	**–**	1.03 (1.02–1.05)	**–**	**–**
Surgery+radiotherapy	1	**–**	3.90 (1.82–8.37)	**–**	**–**
Populations					
<100	6	Random	2.31 (1.22–4.39)	0.000	82.1%
>100	82.1	Random	1.45 (1.21–1.73)	0.025	52.6%

Abbreviations: CRC, colorectal cancer; IGFBP2, insulin‐like growth factor binding protein 2; NOS, Newcastle‐Ottawa Scale; GBM, glioblastoma multiforme; OC, ovarian cancer; OS, overall survival.

### 3.4 Assessment of sensitivity analysis and publication bias

**FIGURE 5:**
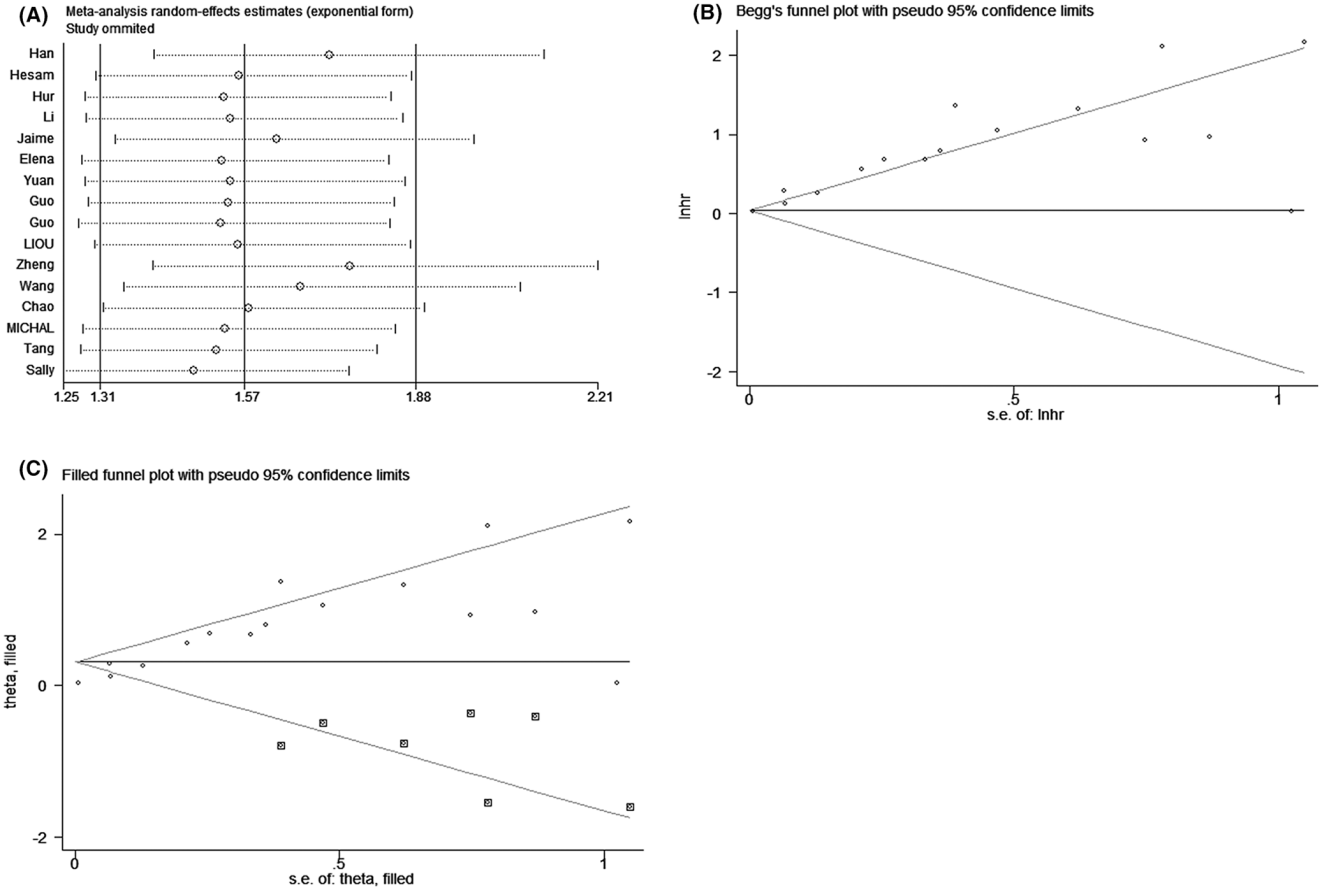
(A) Sensitivity analysis of OS, (B) Begg's funnel plots for publication bias test of OS, (C) Filled funnel plots of OS. Hazard ratios and 95% confidence interval are represented by squares and horizontal line crossing the square, respectively. The diamonds represent the pooled HR and 95% CI. All statistical tests were two‐sided.

The stability of the results was evaluated through sensitivity analysis pooled HRs for OS by eliminating each study, respectively. No significant changes were reported for the pooled HRs. Therefore, the result of the meta‐analysis was proved to be credible (Figure 5A). At the same time, funnel plot and Begg's test were implemented to test for whether there was publication bias (Figure 5B). The results indicated OS had a significant publication bias (*P* < 0.0001). The number of unpublished study assessment was 7 using the trim and fill method as shown in Figure 5C (*P* < 0.0001).

## 4 DISCUSSION

In this meta‐analysis, we conducted the quantitative analysis to evaluate the relationship between IGFBP2 expression and prognosis in various cancer patients. Besides, we performed subgroup analyzes based on ethnicity, cancer type, publication year, NOS score, treatments, and populations. The HR values for OS and PFS were 1.57 (95% CI = 1.31–1.88) and 1.18 (95% CI = 1.04–1.34), respectively, which showed that there was a significantly negative correlation between IGFBP2 expression and OS, PFS in cancer patients. This connection was also observed in both Asians and Caucasians. When analysis was restricted to glioma and colorectal cancer, we found a worse prognosis. Although IGFBP2 expression was related to poor PFS, the result should be interpreted cautiously because of limited included studies. In addition, although IGFBP2 expression was not connected with DFS and RFS, all three articles included in this meta‐analysis for DFS suggested there was a significant connection between IGFBP2 expression and DFS. This may be due to the limited number of articles. Therefore, these results need to be confirmed by more studies.

IGFBP2 is found to be highly expression in many of cancers,^49^, ^50^, ^51^, ^52^ which may emerge as a potential prognostic indicator in various tumors. Moreover, a variety of cellular processes, such as cellular migration, invasion, angiogenesis, epithelial‐to‐mesenchymal transition(EMT), and transcriptional activation,^44^ are related to high IGFBP2 expression in many cancers, thus participating in the development of various cancers. But the potential mechanism of IGFBP2 in cancer is still vague.^53^ Li et al. found that IGFBP2 exert tumor‐promoting functions through a complex regulatory network in tumors.^54^ Hayden et al. discovered that NF‐κB target genes by extensively participating in regulating cell proliferation, apoptosis, and cell migration.^55^ Several studies suggested that IGFBP2 participated in tumorigenesis by multiple potential pathways such as integrin β1/ERK, integrin/ILK/NF‐κB, EGFR/STATA3, and so on.^15^, ^17^, ^56^ Holmes et al. focused that NF‐κB target genes were found in glioma and PDAC, which expressed IGFBP2 steadily.^56^, ^57^ Han et al. demonstrated that chemical resistance and cell proliferation, invasion in glioma patients induced by IGFBP2 by means of the integrin β1/ERK signaling pathway.^17^ The meta‐analysis showed that high IGFBP2 expression is an indicator of poor prognosis in glioma patients. Therefore, it might be an effective treatment for glioma patients by inhibiting IGFBP2 mediating the integrin β1/ERK signaling pathway, in combination with chemotherapy. In addition, Ben‐Shmuel et al found that IGFBP2 promotes colorectal cancer cell progression mediated by the L1/Ezrin/NF‐κB pathway. This suggested inhibition of this pathway might be effective. Chen et al. found that the expression of IGFBP2 boosts acute leukemia cells' survival and migration, as a regulator of the PTEN/AKT pathway.^18^ Dean et al. found that IGFBP2 can be used to indicate the PI3K/AKT pathway activation and PTEN status, and participates in the carcinogens of many types of cancer through the cascade with PTEN.^57^, ^58^, ^59^, ^60^ Therefore, the prognosis of IGFBP2 in underlying mechanism should be explored, and whether pathways of IGFBP2 mediated are helpful in treatment of glioma and colorectal cancer patients needs to be confirmed through more fundamental research.

We also explored the potential source of the high heterogeneity based on PRISMA Guidelines. The subgroup analyses in accordance with ethnicity, publication year, NOS score, treatments, cancer type, and populations were performed. There was still highly significant heterogeneity among 11 studies of Asians, 5 studies of Caucasians, populations >100 and < 100 subgroups. Besides, we found that the heterogeneity decrease in neither before 2016 nor 2016–2021 subgroups. Therefore, ethnicity, publication year, and populations were not the main source of the heterogeneity for OR in this meta‐analysis included 16 studies. We found that the high heterogeneity among included studies reduced obviously according to the treatments. That is to say, treatments were likely to be a source of high heterogeneity. When the analysis was limited to the caner type, we found that the heterogeneity of colorectal cancer decreased obviously. In the same way, the heterogeneity of NOS score <7 also reduced significantly. The heterogeneity could be explained through cancer type and NOS score. The additional source of heterogeneity might be associated with the method of HR and 95% CI obtained from KM curve. We gained HR and 95% CI by Engauge Digitizer and Excel provided by Tierney directly when these data were not reported by the authors.^61^ These studies mainly include several methods such as IHC, ELISA, and RT‐PCR to test the level of the IGFBP2. When IGFBP2 was overexpressed, the cancer patients in these studies had a poor prognosis within all methods. It showed the detection method was unlikely to be the source of heterogeneity.

Several studies related to fields indicated high IGFBP2 expression is correlated with worse prognosis for glioma patients.^24^, ^46^, ^62^ It is consistent with our findings in the subgroup analyses regarding to glioma. The meta‐analysis results provided by Fang et al only reported prognostic value of IGFBP2 expression in glioma.^46^ However, our findings still showed an association between IGFBP2 expression and the prognosis in colorectal cancer. In addition, Gianuzzi at al found that the survival of OC was significantly related to IGFBP2 level, in which they used the mean difference or the standardized mean difference to compare the relationship.^63^ We here used the OR to measure the strength of correlation between IGFBP2 and OC, but a significant connection did not exist in our meta‐analysis. The subgroup analyses for OC consisted of two studies in our meta‐analysis, one of which shows a significant association between IGFBP2 and OC perhaps because of the large sample size gained from GTEx project and TCGA.^36^ The other study had only 99 samples and high IGFBP2 expression was not related to prognosis of OC patients.^35^ Thus, the sample size may be a reason without statistical significance in OC. Besides, limited literature may also lead to the inconsistent results of this study. In addition, we also found that the individual HRs summarized in these forest plots are all larger than one in this meta‐analysis. However, this phenomenon could not directly lead to the conclusion that high IGFBP2 expression is associated with worse outcomes in these patients. As is known to all, meta‐analysis could increase sample size and enhance the statistical power to improve the credibility of the conclusion. We believe that the rigorous statistical analysis performed in this meta‐analysis could provide a more reliable result. Furthermore, many studies from other researchers that carried out meta‐analysis through the similar statistical analysis also support our viewpoint.^64^, ^65^, ^66^, ^67^

The strength of our study was to systematically collect evidence on the prognostic value of IGFBP2 in cancer patients. This makes it possible to use a risk prediction model of including IGFBP2 to forecast prognosis of cancer patients. This meta‐analysis was restricted to the following points. First, some studies only provided KM curve without direct HR and 95% CI, which might influence the result's accuracy. Second, there was significantly high heterogeneity among included studies. Third, this was a retrospective analysis of published studies. As a result, it is vulnerable to these published studies and contributes to several publication bias. Besides, the positive results which tend to be easier to publish is one of the important factors in publication bias, which leads to exaggerating the relationship between IGFBP2 expression and OS.

## 5 CONCLUSIONS

To sum up, the present meta‐analysis shows that there is a significant association between high IGFBP2 expression and worse OS and PFS in various cancer patients. Thus, IGFBP2 may serve as a great biomarker of prognosis in various cancers, and it is worthy of further study to add it to the established risk prediction model to provide clinical the basis for individualized treatment plan. Nevertheless, data are insufficient to determine its role cautiously. More prospective studies of large number of patients are needed to further confirm the value of IGFBP2 in various cancers.

## AUTHOR CONTRIBUTIONS

YWX and YHP contributed to the study design; BZ and CQH searched the literature, BZ and LFW analyzed the data and wrote the manuscript; YHL, YL, and CQH drew the figures and improved the language. All authors read, revised, supervised, and approved the final manuscript.

## FUNDING INFORMATION

This work was supported by funding from the Natural Science Foundation of China (81972801), the Basic and Applied Basic Research Foundation of Guangdong (2019A1515011873), the Li Ka Shing Cross‐Disciplinary Research Grant Foundation (2020LKSFG01B), the Science Medical Project and Shantou Technology Planning (200605115266724), and the Science and Technology Program of Guangdong Esophageal Cancer Institute(Q201906).

## CONFLICT OF INTEREST

The authors declared that there is no conflict of interest for this article.

## ETHICAL DECLARATION

Ethical consent was not required for the systematic review and meta‐analysis as all data obtained from previously published studies.

## Supporting information


**Appendix S1** Supplementary Information

## Data Availability

All data generated or analyzed during this study are included in this published article.
