# Supplementary material for: Prognostic value of IGFBP2 in various cancers: a systematic review and meta‐analysis
Source: Cancer Med. 2022 May 11;11(16):3035–47. doi: 10.1002/cam4.4680 (PMC9385590; doi:10.1002/cam4.4680)
Supplement: Supplementary file 1 — Appendix S1 Supplementary Information [file CAM4-11-3035-s001.zip › Supplementary Materials.pdf]

Supplementary materials

Supplementary Method

The search strategies were as follows: (“Insulin-Like Growth Factor Binding Protein 2” OR “Insulin Like Growth Factor Binding Protein 2” OR “IGFBP-2” OR “IGFBP 2” OR “IGF-Binding Protein 2” OR “IGF Binding Protein 2”) AND (“Neoplasms” OR “Neoplasia” OR “Neoplasias” OR “Neoplasm” OR “Tumors” OR “Tumor” OR “Cancer” OR “Cancers” OR “Malignancy” OR “Malignancies” OR “Malignant Neoplasms” OR “Malignant Neoplasm” OR “Neoplasm, Malignant” OR “Neoplasms, Malignant” OR “Benign Neoplasms” OR “Neoplasms, Benign” OR “Benign Neoplasm” OR “Neoplasm, Benign”) AND (“Prognosis” OR “Prognoses” OR “Prognostic Factors” OR “Factor, Prognostic” OR “Factors, Prognostic” OR “Prognostic Factor” OR “Survival”).

Supplementary figures

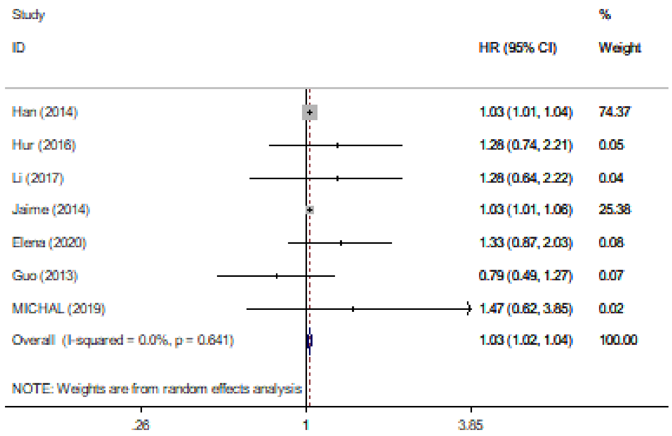

Supplementary Figure 1. Forest plots showing the hazard ratios (HRs) for the cancer risk based on age

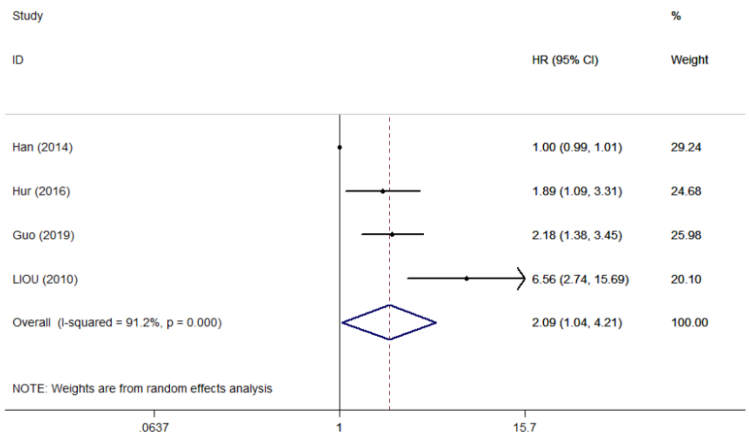

Supplementary Figure 2. Forest plots showing the hazard ratios (HRs) for the cancer risk based on tumor size

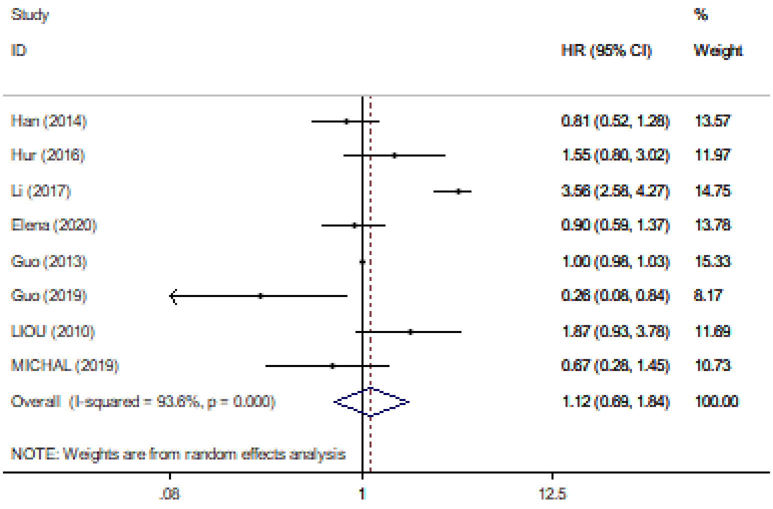

Supplementary Figure 3. Forest plots showing the hazard ratios (HRs) for the cancer risk based on sex
